# Comparison of Flank Super Abrasive Machining vs. Flank Milling on Inconel^®^ 718 Surfaces

**DOI:** 10.3390/ma11091638

**Published:** 2018-09-06

**Authors:** Haizea González, Octavio Pereira, Asier Fernández-Valdivielso, L. Norberto López de Lacalle, Amaia Calleja

**Affiliations:** 1 Department of Mechanical Engineering, University of the Basque Country (UPV/EHU), Plaza Ingeniero Torres Quevedo 1, 48013 Bilbao, Spain; 2 CFAA—University of the Basque Country (UPV/EHU), Parque Tecnológico de Zamudio 202, 48170 Bilbao, Spain; octaviomanuel.pereira@ehu.eus (O.P.); asier.fernandezv@ehu.eus (A.F.-V.); norberto.lzlacalle@ehu.eus (L.N.L.d.L.); 3 Department of Mechanical Engineering, University of the Basque Country (UPV/EHU), Nieves Cano 12, 01006 Vitoria, Spain; amaia.calleja@ehu.eus

**Keywords:** flank super abrasive machining (*SAM*), flank milling, Inconel^®^ 718, roughness, residual stress

## Abstract

Thermoresistant superalloys present many challenges in terms of machinability, which leads to finding new alternatives to conventional manufacturing processes. In order to face this issue, super abrasive machining (SAM) is presented as a solution due to the fact that it combines the advantages of the use of grinding tools with milling feed rates. This technique is commonly used for finishing operations. Nevertheless, this work analyses the feasibility of this technique for roughing operations. In order to verify the adequacy of this new technique as an alternative to conventional process for roughing operations, five slots were performed in Inconel^®^ 718 using flank SAM and flank milling. The results showed that flank SAM implies a suitable and controllable process to improve the manufacture of high added value components made by nickel-based superalloys in terms of roughness, microhardness, white layer, and residual stresses.

## 1. Introduction

Thermoresistant superalloys, such as titanium- and nickel-based alloys, are an actual challenge for manufacturing technologies. These alloys are widely used for many applications that require stability of material properties working under extreme conditions and temperatures up to 400 °C and 600 °C, respectively [1]. One of the main characteristic of these materials, among others, is the optimal combination of hardness and good ductility with low thermal conductivity [2,3]. Superalloys, like Inconel^®^ 718, has multiple applications as a consequence of its mechanical and physical properties, it is spreading to industries such as petrochemical plants, marine equipment, food processing equipment, and nuclear reactors [4]. Nevertheless, these alloys are known as difficult-to-cut materials, implying premature tool wear and high cutting forces [5,6]. Moreover, the challenge lies in the low machinability combined with difficult geometries and finishing requirements that leads to optimizing traditional manufacturing processes, improving cutting strategies, new tools design [7], and cooling techniques on milling Inconel^®^ 718 [8,9].

Nonetheless, traditional methods such as milling, grinding or broaching presents some difficulties due to the low thermal conductivity and extreme high strength of these superalloys [10,11]. An extra critical adverse effect of conventional techniques consists of tool wear; due to the fact that it is conditioned by cutting parameters and cutting fluids [12,13]. For this reason, non-conventional manufacturing technologies were considered as an alternative or complementary, such as electrochemical machining (ECM), linear friction welding (LFW), electro-discharge machining (EDM) [14]. On the other hand, among abrasive machining, it could be found non-conventional technologies, such as abrasive flow machining (AFM), magnetic abrasive finishing (MAF), or magneto-rheological abrasive flow finishing (MRAFF). These processes are known for high surface quality with low-medium removal rates [15,16]. The main advantage of these non-conventional technologies is that they are able to achieve tough dimensional accuracy and excellent finishing surfaces working on complex geometrical cavities where milling has no accessibility [17,18]. However, these technologies offer low material removal rates, translated to higher manufacturing time and costs [19].

In this line, it is important to find more efficient processes that optimize manufacturing time and machining quality. Among these technologies, super abrasive machining (SAM) was presented in [20,21] as a solution to increase machining efficiency during the production of blades and turbine disks [22]. This method consists of applying grinding processes with machining rates and conditions. Besides, under similar cutting conditions of single point machining it offers finishing precisions closest to grinding technology, what makes this process more versatile than grinding or milling techniques. Among grinding techniques with super abrasive grinding tools, it is found the creep feed grinding (CFG) defined as grinding process with larger cutting depths and higher feed rates resulting on higher removal rates decreasing machining time [23]. Petrilli et al. [24] defined creep fatigue grinding as the closest rival for SAM achieving higher speeds and specific material removal rates up to 1000 mm^3^/mm s [20]. Major benefits from SAM reside on higher material removal rates at higher speed along with the near-shape surface obtaining more accurate dimensional tolerances [24,25]. The main limitation for the optimal use of this technology is found in spindle speed requirements, up to 90,000 rpm [26].

**Figure 1:**
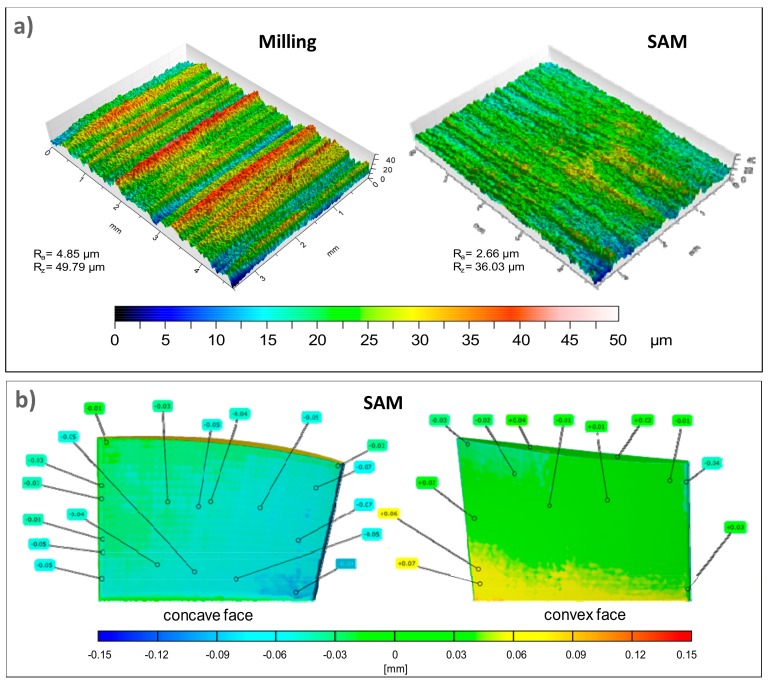
SAM vs. conventional milling obtained results: roughness (**a**) and dimensional deviation (**b**) [27].

Previous work was carried out following this trend, comparing conventional milling with the SAM technique using a conventional machining centre limited to 18,000 rpm of spindle rotary speed. Haizea et al. [27] analysed these techniques, manufacturing a complex geometry made of Inconel^®^ 718, what added an extra challenge related to complex geometry and non-developable surfaces. In terms of surface roughness, dimensional deviation, and force measurement, SAM presented competitive results compared to conventional milling. Figure 1 shows obtained results for roughness and dimensional deviation comparing SAM and milling.

Nevertheless, critical parameters need to be analysed to make this technique a feasible alternative to milling technique or a complementary technique using the same equipment, avoiding clamping and unclamping additional errors, among others. For instance, the appearance of residual stress is considered crucial to be controlled. Some residual stresses lead to plastic deformations or structural modifications [28]. Depending on the industrial applications of some components, compressive residual stresses present improvements on material behaviour; owing to this type of stress prevents from brittle fracture and fatigue failure [29].

Therefore, the novelty of this work stems from the idea of studying the material behaviour about using SAM technology instead of conventional milling technology for roughing using conventional machining centre. This technique is becoming a good alternative for finishing, however this work analyses SAM feasibility for full slot roughing. Based on the concept of multitasking machines, it offers the possibility of applying this technique using conventional machining centres. In order to extend the knowledge about SAM behaviour at roughing operations, five full slots were machined with both techniques (flank SAM and flank milling); selected material was Inconel^®^ 718. In order to analyse the adequacy of flank SAM the following parameters were measured: roughness, surface irregularities, white layer, residual stresses, and microhardness.

## 2. Experimental Setup and Procedure

With the aim of analysing the adequacy of flank SAM compared to flank milling in terms of surface integrity applied to thermo-resistant super alloys, a serial of experiments were designed. The experiments were performed as five full slots with the total effective tool length for flank SAM and for flank milling.

**Figure 2:**
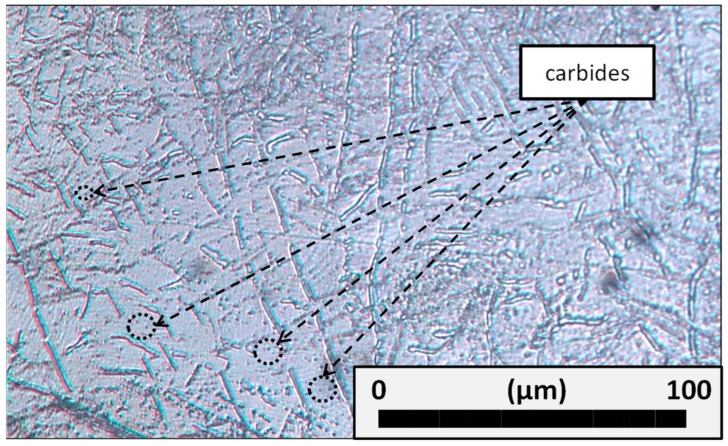
Microstructure of Inconel^®^ 718.

According to previous work, Inconel^®^ 718 was selected as a challenging material for these processes. This material consists of a nickel-based hardened alloy through the precipitation of its metallic matrix secondary phases [30]; Figure 2 shows the microstructure of selected material obtained with an optical microscopy at 50×. It was observed a fine grained structure (ASTM6) with a discrete carbide phase scattered inside the grains, usually presented in Inconel^®^ 718. This superalloy was selected due to the fact that it constitutes one of the most utilized materials in the aeronautic industry, more concretely for engines and turbines [31].

This thermo-resistant super alloy is characterized by good resistance to fatigue and creep combined with high corrosion resistance under extreme working conditions at high temperatures. Nevertheless, it is considered a difficult-to-cut material due to the magnitude of cutting forces, low material removal rates, built-up edges, and extreme tool wear during machining [32,33]. Thus, Inconel^®^ 718 was selected as the material for experimental trials in order to provide data for both manufacturing techniques (flank SAM and flank milling); showing their behaviour working with low-machinable materials and under aggressive machining conditions.

Table 1 shows chemical composition, mechanical and physical properties for the selected material.

**Figure 3:**
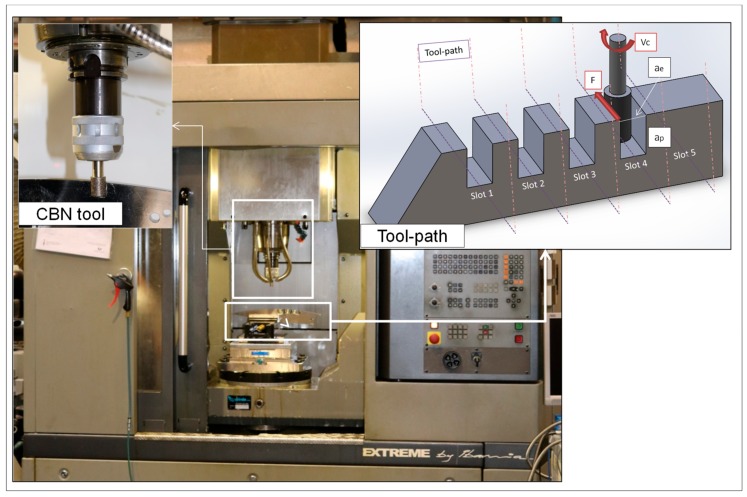
Experimental setup at the University of the Basque Country (UPV/EHU).

Figure 3 shows the experimental set-up for performing defined trials. A five-axis machining centre was used, three linear axes (X, Y, Z) and two rotary axes (A, C). The main limitation of this machine is the spindle speed capacity, a spindle speed up to 18,000 rpm and 18 KW. The machining centre model was an Ibarmia ZV-25/U600 (IBARMIA INNOVATEK, S.L.U., Guipuzkoa, Spain).

Related to manufacturing strategies, flank milling and flank SAM were selected in order to remove the highest amount of material according to tool limitations, using the total cutting effective length. In the case of flank milling, a 16 mm diameter, 20 mm cutting length four-tooth carbide tool coated with AlTiN was selected. This coating was selected because the presence of aluminium implies higher surface hardness and resistance to oxidation [35]. On the other hand, for flank SAM operation a 16 mm diameter, 20 mm cutting length PCBN grinding tool was selected.

Table 2 contains used cutting conditions during trials; cutting parameters and tool type were selected to manufacture Inconel^®^ 718 according to industrial conditions and based on previous experiments [27]. In spite of not using the optimal spindle speed for the SAM technique; in this case, cutting conditions were limited to machine capacities. This leads to the allowance of performing this technique with conventional machining centres.

**Figure 4:**
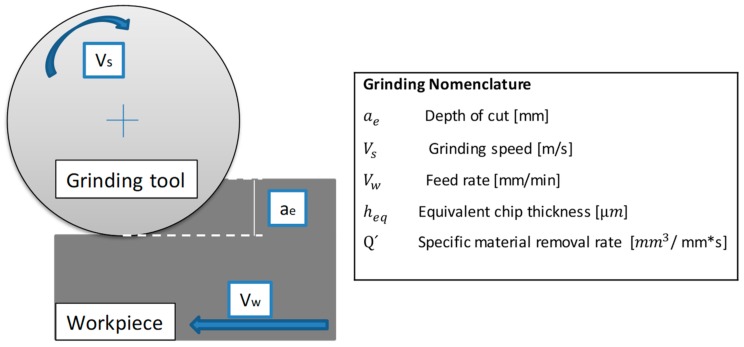
Grinding process parameters definition.

Machine spindle speed led to the use of limited cutting conditions for SAM technology. Though, with the aim of comparing different grinding processes and equate performed processes, there are two parameters commonly used for this purpose inside grinding technology: the equivalent chip thickness (h_eq_) and the specific material removal rate (Q´) [36]. Figure 4 shows the grinding parameters involved in this calculus, followed by the equations.

Q′ = a_e_ v_w_, (1)

(2) $$h_{eq}=a_{e}\;\frac{V_{w}}{v_{s}},$$

Through Equations (1) and (2) equivalent chip thickness and specific material removal rate were calculated. These two concepts depend on the following parameters: cutting depth (a_e_), feed (v_w_), and cutting speed (v_s_). The main difference between the proposed roughing strategies compared with conventional grinding conditions is found in the depth of cut since, in this case, the whole tool diameter was used. Consequently, the equivalent chip thickness obtained was 0.8 µm and the specific material removal rate was 12 mm^3^/mm s. According to Marinescu et al. [37], removal rates in creep feed grinding obtained for Inconel^®^ 989 and Inconel^®^ 718 were around 13 mm^3^/mm s. This process consists of a grinding process characterized by high stock-removal rates with deep depths of cut as the presented case.

## 3. Results and Discussions

With the aim of analysing the feasibility of this manufacturing technique compared to the conventional one, roughness, microstructure and white layer, residual stresses, and microhardness were measured after manufacturing.

### 3.1. Roughness

A Surtronic Duo portable roughness tester from Taylor Hobson^®^ (Ultra Precision Technologies Division of AMETEK Inc., Berwyn, PA, USA) was used to measure roughness during machining trials. It needs to be mentioned that the direction of measurements was perpendicular to the cut. This was carried out in order to obtain the peaks and valleys of the tool teeth, which is the most unfavourable case.

**Figure 5:**
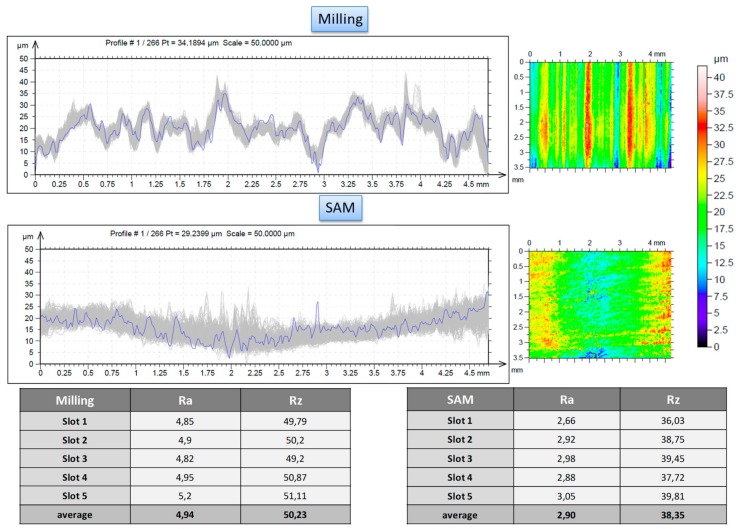
Roughness values (R_a_ and R_z_) for flank milling and flank SAM.

Additionally, after performed tests, a confocal microscope was used to obtain the profile for each sample produced by different techniques. Roughness measuring setting in this case was a 0.8 mm cut-off length and an evaluation length of 4 mm, according to the standard of ISO 4288 [38]. Figure 5 shows obtained data for both the techniques used. Obtained values for flank milling and flank SAM are admissible values for roughing high added-value components in aerospace and aeronautical industries made by this thermo-resistant material [14]. Thus, this implies stable and controllable cutting processes in both cases related to roughness.

Notwithstanding, it should be pointed that under similar cutting conditions, the flank SAM method produces a surface which is characterized by lower value roughness parameters in relation to flank milling, improvements up to 172.28 % and 132.33 % of R_a_ and R_z_, respectively. Moreover, the patterns followed by both processes shown in Figure 3 are typical of milling and grinding processes, respectively. In particular, in the first case the pattern is directional due to the milling tool edge. In the second one, the pattern is non-directional because the grinding tool is composed by small grains which are distributed along the tool surface randomly.

### 3.2. Cross-Section and White Layer

**Figure 6:**
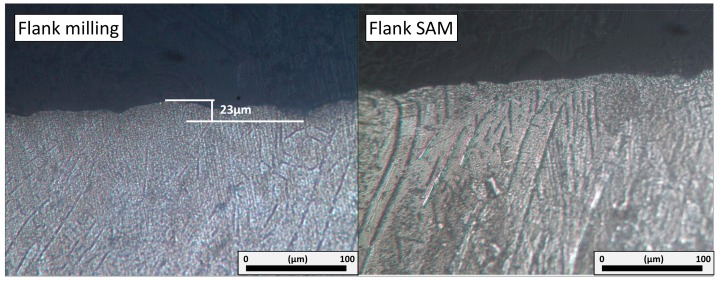
Cross-section of the Inconel^®^ 718 surface after flank milling and flank SAM.

Figure 6 shows the cross-section perpendicular to the workpiece cutting direction for flank milling and flank SAM. Flank milling contained some irregularities on the machined surface with a macroscopic deviation. It is possible that this is as a result of aggressive cutting conditions with thermo-resistant superalloys. Furthermore, as an extra difficulty in the designed tests consisted on opening a full slot, this implied inadequate space for chip removal and refrigeration. Hence, the cutting tool is more prone to suffer from tooth breakage instead of regular wear [39].

On the contrary, the flank SAM top layer presented a regular finished surface with a minimal deviation. This is the consequence of utilizing grinding tools instead of milling tools; these tools have a wear type more regular even in the case of grain detachment [40]. These results explained extensively roughness obtained values. Regarding surface finishing, flank SAM offered a more stable behaviour comparing with conventional technique. This implies the possibility of reducing machining steps in real pieces with the aim of obtaining final surfaces.

The formation of white layer consists of a hard surface layer formed by ferrous materials whilst using high cutting temperatures that can modify the surface integrity. It is produced by a rapid heating during machining over austenitizing temperature and followed by a quick cooling of this surface [41]. Some experts related the existence of this white layer for nickel-based superalloys not only with the high heat during the process but with the low thermal conductivity property of these materials [42]. In order to detect this layer, it is shown in the microscope as a thin white layer. It is important to mention that the existence of the white layer implies a direct discard of technique or conditions if the size overpass is 2 µm [43]. The white layer depends on the cutting parameters and environment contributing to fatigue failure [29].

Chen-Wei Dai et al. [44] found white layer on the surface whilst grinding Inconel^®^ 718 under extreme conditions due to the quick jump from rapid heating to cooling. In this line, considering roughing strategies for flank SAM and flank milling as hard cutting conditions and suffering considerable thermal changes, it is important to point out that no white layer was found for any of these techniques.

### 3.3. Residual Stress

**Figure 7:**
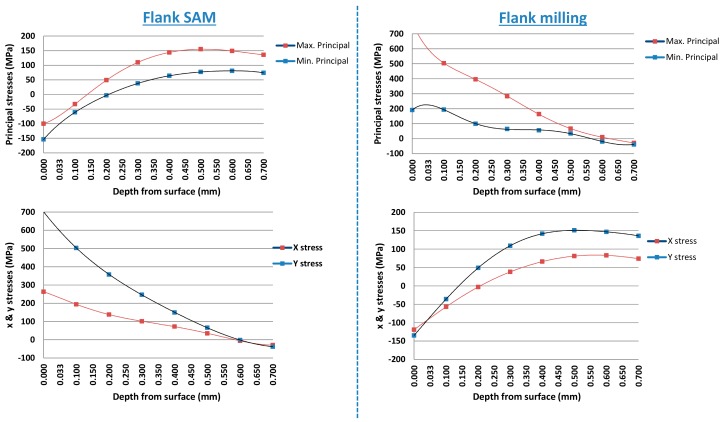
Residual stresses obtained for flank SAM and flank milling on Inconel^®^ 718.

With the aim of measuring residual stress in the final fabricated surfaces and analysing differences between both techniques, hole-drilling strain gage method was selected [45]. According to the selected material, properties for determining residual stresses were established as: Young’s modulus (206 GPa), Poisson’s ratio (0.294), or yield stress (550MPa). The rosette type selected was 062 UL with a mean diameter of 5.13 mm, the hole diameter was 1.94 mm and a limit depth of 0.75 mm. The relation between hole diameter and maximum depth is 0.75/1.94 = 0.386. In line with ASTM standard E837 [45], when applying blind-hole drilling residual stress analysis the relation between hole diameter (D) and maximum hole depth (Z) is specified as Z/D = 0.4. Figure 7 shows the obtained results of principal stresses and each axis stress for flank SAM and flank milling, respectively.

According to residual stresses obtained results, for flank milling technique it was appreciated a tensile residual stress near to machined surface common for this technique [46]. On the contrary, flank SAM measurements showed a compressive pattern residual stress near to the manufactured surface. Additionally to the differences between obtained values magnitude, the most remarkable aspect is that flank SAM values are compressive values, usual for grinding techniques. This implies better behaviour to fatigue failure and prevents brittle fracture.

### 3.4. Microhardness

**Figure 8:**
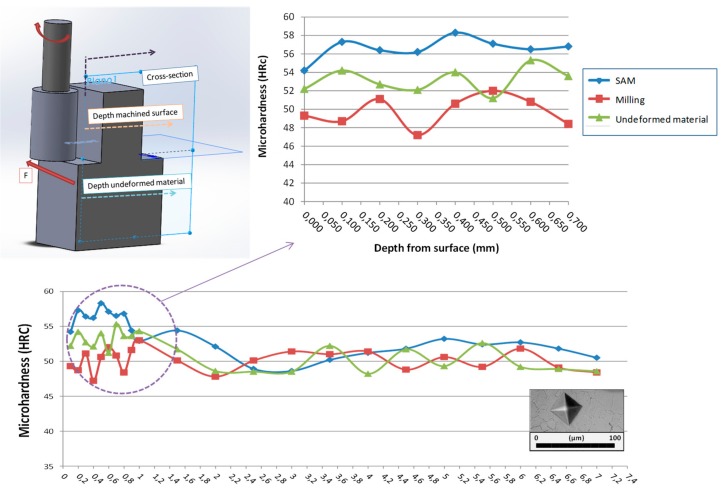
Residual stresses obtained for flank SAM and flank milling on Inconel^®^ 718.

Related to material properties, microhardness was measured for material base, flank milling, and flank SAM. Following the ASTM standard E384 [47] that covers microindentation hardness testing, the Vickers test was selected. Figure 8 shows the Vickers indenter used and the obtained results. According to the ASTM standard E140 [48], these values were converted internally into Rockwell hardness, the more commonly used unit for these materials.

The measuring length along the first 1 mm was 0.1 mm and over this value was 0.5 mm. This was carried out in order to obtain more information close to machined surface. The results showed that undeformed material microhardness presents an average of 52 HRC, with a minimum of 49 HRC and maximum of 55 HRC. These values were set as a reference to study material property variation for both manufacturing techniques. Regarding the machined areas, differences were observed in the first 0.8 mm; approximately the same depth residual stresses were stabilized. In the case of flank milling, microhardness was situated within 46.5 HRC and 53 HRC. Conversely, for flank SAM an increase was observed in microhardness values up to 58 HRC. These values are a direct consequence of the appearance of compressive residual stresses on the machined surface; compressive stresses implied a rise in hardness values. This behavior is in concordance with the study carried out by Hua et al. [49] in which the increase of hardness was related with the maximum hardness value.

## 4. Conclusions

In this work, full slots were manufactured in Inconel^®^ 718 with the main objective of comparing flank milling and flank SAM techniques in conventional machining centers. The new concept of using SAM as a roughing technique apart from finishing strategies leads to considerer this technique as a robust alternative for traditional milling. Additionally, the possibility of adapting a conventional machine to the use of both technologies fits with the trend of multitasking machines.

For this study, both techniques were compared from a technical point of view. In particular, surface roughness, microstructure, white layer, residual stresses, and microhardness were analyzed. The main conclusions obtained are listed below:

- Regarding surface roughness, flank SAM presented lower values for surface roughness comparing with *flank milling*. These results lead to consider flank SAM technique as an alternative for roughing strategies applied to these difficult-to-cut materials. Furthermore, obtained results conclude to the capability of using this technique for replacing intermediate manufacturing stages as semi-finishing strategies.
- Concerning cross-section, it was observed that conventional technique generated irregularities on the machined surface. This is directly related to the differences presented on roughness values. The reason why surface irregularities was avoided with flank SAM derived from the tool type. In this case, the use of grinding tools maintained a more constant tool wear and consequently a regular surface finishing. Additionally, it needs to be pointed that no white layer was found in any case.
- Related to residual stresses, considering the importance of the appearance of residual stress (both tensile and compressive), it should be highlighted that compressive residual stresses and tensile residual stresses arose on the machined surface by flank SAM and flank milling respectively, obtaining values five times higher for the conventional technique. Furthermore, compressive residual stresses in some processes add value to the machined material properties, such as better behavior to fatigue failure.
- Finally, microhardness showed higher values on the flank SAM surface. This improvement of the material property is a direct product of compressive residual stresses.

Therefore, from a technical point of view related with surface integrity in terms of surface roughness, residual stresses, microstructure and microhardness, the results obtained in this experiment showed that flank SAM technology does not present a limitation for being used with conventional machines; as long as cutting conditions were adequately adapted to spindle rotary capacity. This novel technology implies better results than conventional milling obtaining a suitable, controllable and predictable process to manufacture high added value components made of heat-resistant super alloys, such as Inconel^®^ 718.

## Figures and Tables

**Table 1:** Inconel^®^ 718 chemical composition, mechanical and physical properties [34].

**Chemical Composition (%)**												
Ni	Cr	Co	Fe	Nb	Mo	Ti	Al	B	C	Mn	Si	Others
52.5	19	1	17	5	3	1	0.6	0.01	0.08	0.35	0.35	1.79
**Mechanical and Physical Properties**												
Hardness		Young’s Modulus		Tensile Strength		Density		Specific Heat		Melting Temp.		Thermal Conduct
42 HRc		206 GPa		1.73 GPa		8470 kg/m^3^		461 J/(kg·K)		1550 K		15 W/(m·K)

**Table 2:** Defined cutting conditions for the experiments.

Cutting Conditions	Flank Milling	Flank SAM
**Feed Rate**	0.01 mm/tooth	45 mm/min
**Cutting Speed**	20 m/min	900 m/min
**a_p_**	20 mm	20 mm
**a_e_**	16 mm	16 mm
**Cutting Fluid**	Synthetic oil emulsion Houghton^®^ 20%	Synthetic oil emulsion Houghton^®^ 20%
